# Supplementary material for: Effects of preoperative stress, depression, social support, and self-compassion on resilience in surgical patients
Source: PLoS One. 2025 Apr 30;20(4):e0322609. doi: 10.1371/journal.pone.0322609 (PMC12043143; doi:10.1371/journal.pone.0322609)
Supplement: Attachment — Submitted filename: R1_Response to Reviewers.docx [file pone.0322609.s003.docx]

December 20, 2024

Kamalakar Surineni, MD, MPH

Guest Editor

PLOS ONE

Dear Editor and Reviewers:

We would like to thank you for your thoughtful comments and recommendations. We have made the following changes to the manuscript to address your concerns and revised the paper, paying particular attention to language. The revised sections of the manuscript are indicated in RED in the text.

**Reviewer 1**

| **Comments** | **Response** |
| --- | --- |
| 1. The exclusion criteria mentions psychiatric diagnosis such as depression and schizophrenia due to possible reasons of being unable to understand the questionnaire. However it is unclear if any cognitive assessment was done to arrive at this decision. If not, it would be presumptive to assume that psychiatric diagnoses is associated with cognitive impairment which is always not the case. This also limits the ability to generalize findings. | Thank you for the reviewer’s insightful comment regarding the exclusion criteria. To address the reviewer's concerns, we removed the relevant sentence from the exclusion criteria since the inclusion criteria already specified that participants must be able to communicate.  Participants:  The exclusion criteria were as follows: 1) among inpatients, those who were scheduled for surgery with local anesthesia and no anesthesia, and 2) ~~patients with a psychiatric history who were unable to understand the questionnaire items (persons diagnosed with depression and schizophrenia), and 3)~~ emergency surgery patients. |
| 2. Collecting data one day before surgery may not capture the psychological fluctuations that could occur closer to surgery (e.g., weeks before surgery, immediately before, and post-surgery). This does not allow to observe changes in stress and resilience over time. | Based on your recommendations, we have revised the limitation section and added the following points:  Third, the study design is cross-sectional, meaning data was collected at a single point in time. This limits the ability to identify changes in assessments over time. Longitudinal studies would be beneficial for understanding how stress and resilience evolve in the surgical patient |
| 3. Resilience is heavily influenced by cultural contexts. While the tools used are validated, some of the scales (e.g., self-compassion) may not capture all relevant cultural or context-specific nuances. This limitation needs to be added. | As you suggested, we have added more limitation follows:  Finally, resilience is inherently subjective, and its assessment can be influenced by personal and cultural norms. Although some instruments have been validated, individual interpretations may vary. Future research is needed to test reliability and validity in surgical patients, identification of sub-factors through factor analysis, and evaluation of socio-cultural factors from multiple perspectives. |
| 4. The study found no significant correlation between self-compassion and resilience, contrary to expectations based on previous literature. It would be beneficial to explore why this discrepancy occurred, possibly through a deeper analysis of the cultural or contextual factors influencing self-compassion in the study population. | Thank you for your thoughtful review. Based on your recommendations, we have revised the discussion section and added the following points:  Furthermore, the relationship between self-compassion and stress varies across studies. In this study, self-compassion was found to be positively related to depression and surgical stress and negatively related to social support; however, it did not impact resilience. Self-compassion was inversely correlated with isolation and depression among college students during the COVID-19 pandemic [41]. In addition, studies on female adolescents have shown that self-compassion reduces negative emotions, such as depression and anxiety [21,42]. However, previous studies examining different subsets of self-compassion have shown individuals with high levels of self-criticism appear to focus more on their mistakes or failure-related stimuli, which increases their stress and makes them more susceptible to depression [43]. Increasing self-compassion and reducing self-criticism have been suggested as effective ways to overcome these issues. Although this study did not analyze self-criticism and self-compassion separately, patients undergoing surgery are reported to be in stressful situations revealing high levels of self-criticism [30]. Therefore, the correlation between self-compassion and negative emotions such as stress and depression in patients’ preoperative needs to be investigated by categorizing self-compassion into self-compassion and self-criticism through rigorous studies. This observation suggests that although support from family and friends is important before surgery, preventing excessive emotions, such as self-compassion and self-criticism, in patients is vital in increasing resilience. |

**Reviewer 2**

| **Comments** | **Response** |
| --- | --- |
| Abstract: Overall discusses the topic appropriately. However, I would avoid use of embellishments such as the word “basic data” as there is more complexity than that word implies and diminishes the importance of the data. Also, “quick recovery” is mentioned in abstract but is not quite mentioned in the body of the article, so it does not seem to fit. Other grammatical errors, such as past tense should be “data was collected” rather than “data were collected” at various places in the abstract. Also mention how OB surgery affected resilience as results should be included, as current sentence about it seems ambiguous. Another thing that could be considered is putting 4 sections in the abstract, such as background, methods, results, and conclusion to make the abstract more streamlined. Many papers have these sections in abstract to provide more clarity. This is not necessary but can be considered by authors. | As you suggested, we have added a subheading to the abstract for clarity. The revised manuscript was edited by Elsevier Language Editing Services. Certificates of editing have been attached. We hope you find the revised text acceptable.  Abstract  Background  Limited research has been conducted on the psychological factors experienced by patients before surgery. This study examined the effects of preoperative stress, depression, social support, and self-compassion on the resilience of surgical patients.  Methods  Using convenience sampling, 150 patients admitted for surgery at N and S hospitals in I City, South Korea were included in the study. Data were collected from July 1 to 31, 2022. Using SPSS WIN 26.0, the data were analyzed using descriptive statistics, a t-test, ANOVA*, Scheffé’s* test, Pearson’s correlation coefficient, and multiple regression.  Results  Resilience exhibited a statistically significant negative correlation with surgical stress and depression, and a significant positive correlation with social support. Higher education level, economic status, lower surgical stress, depression, and gynecologic surgery were found to influence surgical patients' resilience.  Conclusion  Enhancing resilience in surgical patients requires the development of tailored interventions to mitigate surgical stress and depression, which are patient-centered and account for educational and economic status, particularly in obstetric and gynecological surgery. |
| Introduction: There is good information in this section about prior research studies on this topic and the need for a new research question. There are many grammatical errors in the writing, however, that need revision. For example, the first sentence in this section is not a complete sentence. On page 3, the sentence “The meta-analysis suggested that…” is a run-on sentence and grammar needs revision. Same with next sentence “In addition, resilience…” In the prior paragraph, “emotional confusion” is mentioned but it is not clear what this means and how it’s supported by research. In the last para, it mentions “Patients undergoing surgery also experience severe pressure and depression…” The word “severe” seems like an embellishment and absolute to all patients undergoing surgery, which is not clearly shown how it is supported by research. The next sentence starts with “However, recent studies of surgical stress…” The “however” word does not seem to fit as it does not seem to relate clearly in that way to the previous sentence. There are other overall grammatical errors in the introduction that requires more work. | As suggested by the reviewer and to improve readability and clarity. The corresponding changes have been reflected in the text, highlighted in red (Introduction).  A meta-analysis suggested that higher levels of resilience are associated with faster and more effective recovery from stressful experiences, protection of mental health, and mitigation of the negative effects of a crisis [6], all of which positively affect physical recovery after surgery [7]. In addition, resilience may protect patients with cancer from emotional distress [8] and have a positive effect on self-care behavior [9]. Resilience can be an important factor in overcoming stressful events of surgery as it affects the physical and contributes to maintaining better health. Therefore, in surgical nursing, it is important to identify and enhance the factors that influence resilience.  In a meta-analysis of resilience, stress was found to be negatively correlated [10]. Stress poses a threat to physical well-being; moreover, surgery is accompanied by physical discomfort and psychological burden [4]. Patients undergoing surgery often experience intense pressure and depression due to uncertainty about the procedure, fear of anesthesia-related death, and concerns about postoperative pain or complications, all of which create significant stress for both patients and their families [4]. Nevertheless, recent studies on surgical stress have primarily focused on specific patient groups or patients with cancer, or have measured surgical stress solely using a stress thermometer (100 mm VAS) [11,12]. |
| Methods:  The methods are described reasonably well. It is well-presented with different headings and each parameter is discussed separately (such as self-compassion), which is great. There are again English errors that need correction. On page 6, first paragraph again needs major revisions in the English sentence structure and grammar. There is a flipping between past and present tense as well, which would need change and kept consistent instead. On page 7, the last line is “To measure self-compassion…” This is not a complete sentence. | The methods section has once again been carefully revised, including English sentence structure and grammar.  Data collection  Data were collected from N and S Hospitals in I City, Korea, between July 1 and 31, 2022. After receiving permission from both hospitals, the researchers visited the patients one day before surgery. Data were collected after the participants were informed of the purpose of the study and provided written informed consent. The participants were asked to complete the questionnaires independently; however, if they encountered difficulties, the data collectors read the questions aloud and marked their responses. The participants required approximately 30 minutes to complete the questionnaires and were subsequently presented a gift.  Self-compassion was assessed using the Korean version of the self-compassion scale [27]. |
| Results:  Overall well presented with good use of tables. Sentence structure again needs work at some places. For example, on page 9 it states “The 39 patients were in 40s…” does not require “The” word and can be presented better. On page 11, it would be helpful for authors to describe what these values mean subjectively when interpreted. For example, was the total score of surgical stress represent overall moderate level of stress, or low or high, etc.? This would make the article easier to follow for the reader. On page 12, it states “self-compassion was significantly positively correlated with surgical stress…”. This suggests that higher self-compassion means more surgical stress, which is counter-intuitive. Please clarify potential reasons for this, or correct if it was an error. Later in that page, there is a mention of “self-regret” which is not explained anywhere in the article. Only self-compassion was explained. Later it also mentions “self-disappointment”. Authors should avoid introducing new variables that have not been explained earlier. | Thank you for your thoughtful review. As you suggested, we have unified the term self-compassion and made the following changes to improve clarity.  ~~The~~ Thirty-nine patients (26.0%) were in their 40s, and the mean score for resilience was 91.18±12.62.  The average score for surgical stress was 59.61 out of 140 points, indicating a moderate level of stress. The sub-factors had average scores of 27.53 points for physical factors (moderate), 17.52 points for emotional factors (low), and 14.56 points for environmental factors (low). The total score for depression was 33.97 80, indicating moderate depression. The average score social support was 5.47 out of 6, indicating a high support. The total score for self-compassion was 72.62 out of 130, indicating a moderate self-compassion. The sub-factors of self-compassion were common humanity (12.37, moderate), isolation (9.55, low), mindfulness (13.19, moderate), and over-identification (10.09, low). The total resilience score was 87.57 out of 125, indicating a moderate level of resilience (Table 2).  Self-compassion was significantly positively correlated with surgical stress (r=.293, *p*<.001) and depression (r=.278, *p*<.001) and negatively correlated with social support (r=-.194, *p*<.05). |
| Discussion: Overall this was a section that discussed the results and limitations well. Again, it mentions self-regret which is not clarified or explained earlier in the article. On page 14, the sentence “We also found that patients who underwent surgery showed high social support was.” Is not a correct sentence. On page 15, it mentions colorectal cancer and it is being made parallel to gynecological surgery, when they are very different. Later there is a mention of “special calculations” and unclear what this means. On page 16, the sentence “However, prior education on surgery methods…” is a run-on sentence. There are grammatical errors on this page. The discussion also does not include self-compassion which was one key parameter. | Thank you for your thoughtful review. Based on your recommendations, we have revised the discussion section and added the following points:  We also observed that patients who underwent surgery had a high social support score of 5.47.  However, providing preoperative education and preparation of patients and their families—who constitute the primary social network— as along with educating them on post-discharge patient management, will reduce uncertainty regarding the surgical process and alleviate depression by fostering a sense of security.  Furthermore, the relationship between self-compassion and stress varies across studies. In this study, self-compassion was found to be positively related to depression and surgical stress and negatively related to social support; however, it did not impact resilience. Self-compassion was inversely correlated with isolation and depression among college students during the COVID-19 pandemic [41]. In addition, studies on female adolescents have shown that self-compassion reduces negative emotions, such as depression and anxiety [21,42]. However, previous studies examining different subsets of self-compassion have shown individuals with high levels of self-criticism appear to focus more on their mistakes or failure-related stimuli, which increases their stress and makes them more susceptible to depression [43]. Increasing self-compassion and reducing self-criticism have been suggested as effective ways to overcome these issues. Although this study did not analyze self-criticism and self-compassion separately, patients undergoing surgery are reported to be in stressful situations revealing high levels of self-criticism [30]. Therefore, the correlation between self-compassion and negative emotions such as stress and depression in patients’ preoperative needs to be investigated by categorizing self-compassion into self-compassion and self-criticism through rigorous studies. This observation suggests that although support from family and friends is important before surgery, preventing excessive emotions, such as self-compassion and self-criticism, in patients is vital in increasing resilience. |
