# Supplementary material for: Effects of preoperative stress, depression, social support, and self-compassion on resilience in surgical patients
Source: PLoS One. 2025 Apr 30;20(4):e0322609. doi: 10.1371/journal.pone.0322609 (PMC12043143; doi:10.1371/journal.pone.0322609)
Supplement: Attachment — Submitted filename: Revised Manuscript.docx [file pone.0322609.s002.docx]

**Effects of preoperative stress, depression, social support, and self-compassion on resilience in surgical patients**

Heejoung Lee^1^, Hana Ko^2,*^

^1^ Nasaret International Hospital, Incheon, South Korea

^2^ College of Nursing, Gachon University, Incheon, South Korea

*** Correspondence**

E-mail: hanago11@gachon.ac.kr

**Abstract**

**Background**

Limited research has been conducted on the psychological factors experienced by patients before surgery. This study examined the effects of preoperative stress, depression, social support, and self-compassion on the resilience of surgical patients.

**Methods**

Using convenience sampling, 150 patients admitted for surgery at N and S hospitals in I City, South Korea were included in the study. Data were collected from July 1 to 31, 2022. Using SPSS WIN 26.0, the data were analyzed using descriptive statistics, a t-test, ANOVA, *Scheffé’s* test, Pearson’s correlation coefficient, and multiple regression.

**Results**

Resilience exhibited a statistically significant negative correlation with surgical stress and depression, and a significant positive correlation with social support. Higher education level, economic status, lower surgical stress, depression, and gynecologic surgery were found to influence surgical patients' resilience.

**Conclusion**

Enhancing resilience in surgical patients requires the development of tailored interventions to mitigate surgical stress and depression, which are patient-centered and account for educational and economic status, particularly in obstetric and gynecological surgery.

**Introduction**

With increased public interest in health and advancements in medical technology and surgical techniques, the number of patients visiting medical institutions is increasing. In 2021, the number of patients undergoing surgical treatment in South Korea will increase, with 1,493,000 adult surgical patients accounting for 24% of adult inpatients (6,176,000) and 97% of all surgical patients (1,534,000) [1].

Patients scheduled for surgery experience various negative emotional reactions, such as activity restrictions, socioeconomic burden, apprehension regarding postoperative sequelae, and deterioration in physical function [2,3]. Undergoing a surgical procedure, even a minor one, is perceived as major events that cause discomfort and anxiety [3,4]. Such negative emotional reactions can impact the course of surgery and recovery [4].

Resilience is defined as the capacity to overcome stress or adverse experiences [5]. A meta-analysis suggested that higher levels of resilience are associated with faster and more effective recovery from stressful experiences, protection of mental health, and mitigation of the negative effects of a crisis [6], all of which positively affect physical recovery after surgery [7]. In addition, resilience may protect patients with cancer from emotional distress [8] and have a positive effect on self-care behavior [9]. Resilience can be an important factor in overcoming stressful events of surgery as it affects the physical and contributes to maintaining better health. Therefore, in surgical nursing, it is important to identify and enhance the factors that influence resilience.

In a meta-analysis of resilience, stress was found to be negatively correlated [10]. Stress poses a threat to physical well-being; moreover, surgery is accompanied by physical discomfort and psychological burden [4]. Patients undergoing surgery often experience intense pressure and depression due to uncertainty about the procedure, fear of anesthesia-related death, and concerns about postoperative pain or complications, all of which create significant stress for both patients and their families [4]. Nevertheless, recent studies on surgical stress have primarily focused on specific patient groups or patients with cancer, or have measured surgical stress solely using a stress thermometer (100 mm VAS) [11,12].

Depression has been reported to decrease survival rates [13] and affect resilience in patients with colorectal cancer [11]. Moreover, patients undergoing orthopedic surgery, who require muscle recovery exercises after surgery, the occurrence of depressive symptoms negatively affects postoperative recovery [14-16]. Nursing interventions that continuously assess and manage resilience-related depression should be implemented to improve the prognosis of surgical patients [17].

Social support is provided by family, relatives, neighbors, and friends, upon whom an individual can rely for caregiving and from whom they can derive a sense of value [18]. Higher levels of social support are associated with increased postoperative psychosocial functioning, quality of life, and surgical recovery [11, 19] underscoring the significance of social support. Furthermore, social support demonstrates a positive correlation with the resilience of patients who have undergone surgical procedures [7, 20].

Self-compassion promotes adaptability and resilience in addressing life challenges and represents a crucial construct in mental health and psychological research [21,22]. High levels of self-compassion are associated with a reduced risk of psychopathology and improved resilience [23].

As indicated by previous research, the relationship between each variable has been examined; however, there is a paucity of studies that have comprehensively investigated these relationships. Research on preoperative psychological factors in surgical patients remains insufficient.

Consequently, this study aimed to elucidate the effects of preoperative stress, depression, social support, and self-compassion on resilience in surgical patients to 1) determine the degree and differences in general characteristics, preoperative stress, depression, social support, self-compassion, and resilience among surgical patients; 2) examine the correlations between preoperative stress, depression, social support, self-compassion, and resilience in surgical patients; and 3) determine the impact of preoperative stress, depression, social support, and self-compassion on the resilience of surgical patients.

**Methods**

**Participants**

The participants of this study were patients admitted for surgery to N and S general hospitals located in City I. The selection criteria included: 1) adult surgery patients over 20 years of age; 2) patients who were able to communicate, understand, and respond to the contents of the questionnaire; 3) patients without sensory impairment such as hearing and language impairment; and 4) inpatients scheduled for surgical procedures with anesthesia (general, spinal, and brachial plexus anesthesia). The exclusion criteria were as follows: 1) among inpatients, those who were scheduled for surgery with local anesthesia and no anesthesia, and 2) emergency surgery patients.

The sample size required for multiple regression analysis was calculated using G*Power 3.1 (Heinrich-Heine-University, Düsseldorf, Germany), with a significance level of 0.05, an explanatory power of 0.80, and an intermediate effect size of 0.16 [17]. The required sample size for the regression analysis with 11 independent variables was 110 samples. We analyzed all 150 questionnaires completed by participants who consented to participate in the study.

**Data collection**

Data were collected from N and S Hospitals in I City, Korea, between July 1 and 31, 2022. After receiving permission from both hospitals, the researchers visited the patients one day before surgery. Data were collected after the participants were informed of the purpose of the study and provided written informed consent. The participants were asked to complete the questionnaires independently; however, if they encountered difficulties, the data collectors read the questions aloud and marked their responses. The participants required approximately 30 minutes to complete the questionnaires and were subsequently presented a gift.

**Measures**

**Sociodemographic characteristics**

General characteristics (e.g., age, sex, marital status, religion, occupation, and economic status) were self-reported. Surgery type was categorized as general (appendectomy and pancreatic resection), obstetric and gynecological (hysterectomy and ovarian removal), orthopedic (tendon repair, open and non-open reduction fixation, and artificial joint surgery), and other surgeries (urethral stone removal, incision and biopsy, pin removal, scrotal hydrocele removal, varicose vein removal, carpal tunnel syndrome surgery, and skin grafting).

**Surgical stress**

Surgical stress was measured using a stress measurement tool [24] to assess the emotional state of patients in the preoperative stage. This tool consists of 28 items: twelve surgery-related physical factors, nine surgery-related emotional factors, and seven surgery-related environmental factors. Each item was scored on a 5-point Likert scale, with higher scores indicating higher stress levels. The Cronbach’s α of this scale at the time of its development was .92, and it was .96 in this study.

**Depression**

Depression was assessed using the Korean version of the Center for Epidemiological Studies Depression Scale (CES-D scale) [25]. It consists of 4 positive and 16 negative items, totaling 20 items. For each item regarding how a patient felt during the past week, “rarely (less than 1 d)” scored 1 point, “sometimes (2-3 days)” 2 points, “very much (4-5 days)” 3 points, and “mostly (6 -7 days)” 4 points. A higher final score, derived by inversely converting the four positive questions, indicates a greater degree of depression. The Cronbach’s α of this scale at the time of its development and in this study was .87 and .91, respectively.

**Social support**

Social support was measured using the Korean version of the Enhancing Recovery in Coronary Heart Disease (ENRICHD) Social Support Instrument (ESSI) [26]. The ES-SI consists of six items, including emotional, informational, and instrumental support, and responses of “yes” or “no” were assigned to each item. In the case of a “yes” response to each item, 1 point was given, and the total score was classified as “poor (3 points or less),” “normal (4 to 5 points),” and “good (6 or more points).” Higher total scores indicate better social support. The Cronbach’s α of this scale was .84 and .87 at the time of its development and in this study, respectively.

**Self-compassion**

Self-compassion was assessed using the Korean version of the self-compassion scale [27]. The Self-Compassion Scale consists of three opposing pairs and six sub-factors: self-kindness, self-judgment, common humanity, isolation, mindfulness, and over-identification. A total of 26 items are scored on a 5-point scale, with total scores ranging from 26 to 130 points. Higher scores indicate more self-compassionate attitudes. The Cronbach’s α of this scale at the time of its development was .93 and was .85 in this study.

**Resilience**

The Korean version of the Connor-Davidson Resilience Scale was used [28] to measure resilience. It comprises five factors: tenacity, continuity/endurance, optimism, support, and spirituality. It includes a total of 25 items: 0 points for “not at all,” 1 point for “disagree,” 2 points for “normally,” 3 points for “mostly so,” and 4 points for “very much so,” with a score range of 0 to 100 points. Higher values indicate higher resilience. The Cronbach’s α of this scale at the time of its development was .89 and was .95 in this study.

**Ethical considerations**

This current study was reviewed and approved by the Institutional Review Board of Gachon University (approval no.1044396-202206-HR-125-01). Written consent to participate in the study was obtained from the research participants one day before surgery. We informed participants that they could withdraw from the study at any time. To ensure the confidentiality of their personal information, completed questionnaires were placed in sealed, opaque envelopes, and each questionnaire was assigned a unique identification number to prevent identification during data analysis. The questionnaires were stored in a locked, designated box, accessible only to the researcher directly involved in the study. The research database was securely stored on a computer with restricted access to safeguard it from external exposure during document preparation and data management.

**Data analysis**

The data collected in this study were analyzed using IBM SPSS version 26.0. All data are presented as descriptive statistics, including means and standard deviations. Differences between the general characteristics and resilience of the participants were analyzed using an independent t-test and one-way analysis of variance (ANOVA), and the *Scheffé* test was used for the post hoc test. Skewness and kurtosis were calculated to determine whether the assumptions of normality of the variables were satisfied. Correlations between preoperative stress, depression, social support, self-compassion, and resilience were analyzed using Pearson’s correlation coefficients. Hierarchical multiple linear regression analysis was used to determine whether participants’ preoperative stress, depression, social support, and self-compassion affected their resilience.

**Results**

**Resilience according to the sociodemographic characteristics of the surgical patients**

The patients’ sociodemographic data are presented in Table 1. Of the 150 participants, 85 (56.7%) were men, and the mean score for resilience was 89.66±14.31. Thirty-nine patients (26.0%) were in their 40s, and the mean score for resilience was 91.18±12.62. There were statistically significant differences in resilience according to educational level (F=7.153, *p*=.001), living arrangements (F=4.238, *p*=.016), employment status (t=2516, *p*=.016), economic status (F=8.630, *p*<.001), and type of surgery (F=5.366, *p*=.002).

**Table 1.** Resilience values of the participants by sociodemographic characteristics (N=150)

| **Characteristics** | **Category** | **N(%)** | **Resilience values** | | | |
| --- | --- | --- | --- | --- | --- | --- |
|  |  |  | **Mean(SD)** | **t or F** | **p** | **Scheffé** |
| Sex | Male | 85 (56.7) | 89.66 (14.31) | 1.973 | .050 |  |
|  | Female | 65 (43.3) | 84.85 (15.44) |  |  |  |
| Age | 20's | 27 (18.0) | 90.41 (16.74) | 2.352 | .057 |  |
|  | 30's | 21 (14.0) | 86.10 (15.35) |  |  |  |
|  | 40's | 39 (26.0) | 91.18 (12.62) |  |  |  |
|  | 50's | 37 (24.7) | 87.43 (10.86) |  |  |  |
|  | 60+ | 26 (17.3) | 80.62 (18.95) |  |  |  |
| Educational level | Junior high school^a^ | 19 (12.6) | 77.21 (18.45) | 7.153 | .001 | a<b,c |
|  | High school^b^ | 59 (39.3) | 86.68 (13.89) |  |  |  |
|  | $\geq$College^c^ | 72 (48.0) | 91.04 (13.55) |  |  |  |
| Marital status | Married^a^ | 90 (60.0) | 87.93 (12.68) | 4.238 | .016 | c<a,b |
|  | Unmarried^b^ | 46 (30.7) | 90.04 (16.57) |  |  |  |
|  | Other (separation, divorce, bereavement, etc.)^c^ | 14 (9.3) | 77.14 (19.24) |  |  |  |
| Religion | Buddhism | 10 (6.7) | 91.10 (16.62) | 2.607 | .054 |  |
|  | Christian | 30 (20.0) | 81.07 (19.56) |  |  |  |
|  | Catholic | 24 (16.0) | 90.50 (17.17) |  |  |  |
|  | Other(none) | 86 (57.3) | 88.62 (11.50) |  |  |  |
| Employment status | Employed | 117 (78.0) | 89.51 (13.09) | 2.516 | .016 |  |
|  | Unemployed | 33 (22.0) | 80.70 (18.89) |  |  |  |
| Economic status | Low^a^ | 21 (14.0) | 76.38 (16.07) | 8.630 | <.001 | a<b,c |
|  | Middle^b^ | 117 (78.0) | 88.80 (13.68) |  |  |  |
|  | High^c^ | 12 (8.0) | 95.17 (16.34) |  |  |  |
| Type of surgery | General surgery^a^ | 26 (17.4) | 85.46 (18.44) | 5.366 | .002 | b<a,c,d |
|  | Obstetrics and Gynecology surgery^b^ | 12 (8.0) | 72.58 (20.78) |  |  |  |
|  | Orthopedic surgery^c^ | 61 (40.7) | 89.52 (12.36) |  |  |  |
|  | Others(urethral stone removal, incision and biopsy, pin removal, etc.)^d^ | 51 (34.0) | 89.84 (12.31) |  |  |  |

**Degree of stress, depression, social support, self-compassion, and resilience before surgery**

The average score for surgical stress was 59.61 out of 140 points, indicating a moderate level of stress. The sub-factors had average scores of 27.53 points for physical factors (moderate), 17.52 points for emotional factors (low), and 14.56 points for environmental factors (low). The total score for depression was 33.97 80, indicating moderate depression. The average score social support was 5.47 out of 6, indicating a high support. The total score for self-compassion was 72.62 out of 130, indicating a moderate self-compassion. The sub-factors of self-compassion were common humanity (12.37, moderate), isolation (9.55, low), mindfulness (13.19, moderate), and over-identification (10.09, low). The total resilience score was 87.57 out of 125, indicating a moderate level of resilience (Table 2).

**Table 2.** Degrees of stress, depression, social support, self-compassion, and resilience before surgery (N=150)

| **Variable** | **Categories** | **Range** | **Mean (SD)** | **Skewness** | **Kurtosis** |
| --- | --- | --- | --- | --- | --- |
| Surgical stress | Physical component | 12-54 | 27.53 (9.68) | 0.54 | -0.46 |
|  | Emotional component | 8-38 | 17.52 (7.15) | 0.77 | 0.10 |
|  | Environmental factors | 7-33 | 14.56 (5.39) | 0.83 | 0.71 |
|  | Total score | 28-124 | 59.61 (20.91) | 0.69 | 0.00 |
| Depression | Total score | 20-69 | 33.97 (8.84) | 0.99 | 1.00 |
| Social support | Total score | 0-6 | 5.47 (1.34) | -2.89 | 7.96 |
| Self-compassion | Self-kindness | 7-25 | 15.42 (2.81) | -0.01 | 1.24 |
|  | Self-judgment | 6-25 | 12.00 (3.16) | 0.69 | 1.33 |
|  | Common humanity | 4-20 | 12.37 (2.83) | -0.16 | 0.49 |
|  | Isolation | 4-20 | 9.55 (2.64) | 0.54 | 1.00 |
|  | Mindfulness | 4-20 | 13.19 (2.55) | -0.57 | 0.90 |
|  | Over-identification | 4-20 | 10.09 (2.69) | 0.43 | 0.33 |
|  | Total score | 36-130 | 72.62 (10.83) | 0.40 | 5.36 |
| Resilience | Total score | 25-125 | 87.57 (14.95) | -0.38 | 1.80 |

**Correlation between stress, depression, social support, self-compassion, and resilience before surgery**

Resilience was significantly negatively correlated with surgical stress (r=-.356, *p*<.001) and depression (r=-.452, *p*<.001) and significantly positively correlated with social support (r=.354, *p*<.001). Self-compassion was significantly positively correlated with surgical stress (r=.293, *p*<.001) and depression (r=.278, *p*<.001) and negatively correlated with social support (r=-.194, *p*<.05). Social support was significantly and negatively correlated with surgical stress (r=-.417, *p*<.001) and depression (r=-.529, *p*<.001). Depression showed a statistically significant positive correlation with surgical stress (r=.655, *p*<.001) (Table 3).
**Table 3.** Correlations among preoperative stress, depression, social support, self-compassion, and resilience (N=150)

|  | **Surgical stress** | **Depression** | **Social support** | **Self-compassion** | **Resilience** |
| --- | --- | --- | --- | --- | --- |
| Surgical stress | 1 |  |  |  |  |
| Depression | .655*** | 1 |  |  |  |
| Social support | -.417*** | -.529*** | 1 |  |  |
| Self-compassion | .293*** | .278*** | -.194* | 1 |  |
| Resilience | -.356*** | -.452*** | .354*** | -.140 | 1 |

* p<.05 ** p<.01 *** p<.001

**Factors affecting the resilience of patients before surgery**

Hierarchical multiple linear regression analysis was conducted to examine the effects of preoperative stress, depression, social support, and self-compassion on resilience. In the case of self-compassion, no correlation was found with the results of this study. However, based on previous studies, it was included, and its effect on resilience was examined. The demographic and sociological background variables that were found to be significantly related to resilience through a difference test, including educational level, marital status, employment status, economic status, and surgery type, were controlled for. The VIF index was less than 10 (1.203–2.225), which did not cause multicollinearity, and the Durbin-Watson coefficient was 1.958, which was close to 2, thus satisfying the assumption of residual independence.

In Model 1, control variables such as educational level, marital status, employment status, economic status, and surgery type were treated as dummy variables and inputs. In Model 2, surgical stress, depression, social support, and self-compassion were used as inputs to examine their effects on resilience.

First, in Model 1, in which the control variables were input, economic status (β=.242, *p*<.01) and Obstetrics & gynecological surgery (β=-.251, *p*<.01) were found to have a significant effect on resilience and appeared to have an explanatory power of 18.9% (F=5.350, *p*<.001).

As a result of the additional inputs of surgical stress, depression, social support, and self-compassion in Model 2, the explanatory power increased by 14.7% compared to Model 1, resulting in a total explanatory power of 32.6% (F=6.996, *p*<.001). Surgical stress (β=-.185, *p*<.05) and depression (β=-.204, *p*<.05) had significant negative effects on resilience, whereas educational level (β=.183, *p*<.05), economic status (β=.191, *p*<.05), and Obstetrics & gynecological surgery (β=-.173, *p*<.05) affected resilience (Table 4).

**Table 4.** Effects of preoperative stress, depression, social support, and self-compassion on resilience (N=150)

| **Predictors** | **Model 1** | | | | **Model 2** | | | |
| --- | --- | --- | --- | --- | --- | --- | --- | --- |
|  | **B** | **β** | **t** | **p** | **B** | **β** | **t** | **p** |
| Educational level | 2.707 | .126 | 1.404 | .163 | 3.938 | .183 | 2.162 | .032 |
| Married | -1.828 | -.060 | -0.725 | .470 | -0.387 | -.013 | -0.166 | .868 |
| Employed | 0.976 | .027 | 0.309 | .758 | -0.682 | -.019 | -0.235 | .815 |
| Economic status | 7.748 | .242 | 2.990** | .003 | 6.130 | .191 | 2.560 | .012 |
| General surgery | -5.166 | -.131 | -1.587 | .115 | -3.176 | -.081 | -1.051 | .295 |
| Obstetrics and Gynecology surgery | -13.779 | -.251 | -3.061 | .003 | -9.524 | -.173 | -2.268 | .025 |
| Orthopedic surgery | 0.833 | .027 | 0.323 | .747 | 2.275 | .075 | 0.948 | .345 |
| Surgical stress |  |  |  |  | -0.132 | -.185 | -1.987* | .049 |
| Depression |  |  |  |  | -0.344 | -.204 | -2.030* | .044 |
| Social support |  |  |  |  | 0.978 | .088 | 1.071 | .286 |
| Self-compassion |  |  |  |  | 0.024 | .017 | 0.230 | .818 |
| R² (adjR²) | .233 (.189) | | | | .380 (.326) | | | |
| F | 5.350 | | | | 6.996 | | | |
| P | <.001 | | | | <.001 | | | |

Note: Marital status (not married=0), Employment (unemployed=0), Surgery (Others=0)

**Discussion**

This descriptive study analyzed the effects of preoperative stress, depression, social support, and self-compassion on resilience in surgical patients to provide basic data for comprehensive and personalized nursing support to aid rapid recovery after surgery.

The mean preoperative stress score was 59.61, and the mean depression score was 33.97, supporting the findings of a previous study [24] that surgical patients experienced moderate stress and depression. Patients who are about to undergo surgery, regardless of the extent of the surgery, experience high levels of stress and depression owing to anxiety about the surgery and concerns regarding the outcomes.

We also observed that patients who underwent surgery had a high social support score of 5.47. This finding may be attributed to the fact that social support increases when individuals experience stressful situations, during illness or crises, or those involving conflict, frustration, or changes requiring adjustment [29]. Therefore, interventions are needed to assist individuals with high levels of social support in buffering against stressful events.

After identifying the factors influencing patients' resilience before surgery, the explanatory power was found to be 32.6%. Factors such as degree of education, economic status, surgery type (obstetrics and gynecology), surgical stress, and depression had a significant effect on resilience. The results of this study are consistent with those of other studies [11], which reported that the site of surgery impacts resilience and that the presence or absence of a stoma after colorectal cancer surgery also affects resilience. The findings of this study indicate that the type of surgery, even if considered uncomplicated, can significantly affect patient resilience. In addition, gynecological surgery patients showed lower resilience than other patients [30]. Obstetrics and gynecology surgeries are often viewed as procedures involving organs that represent key aspects of womanhood, such as maternal love, sexual attraction, and femininity [17,31]. These results underscore the importance of implementing interventions to enhance resilience, particularly for patients undergoing gynecological surgery.

Economic status also appears to affect resilience. This finding suggests that economic conditions can increase resilience [11]. In Korea, patients with cancer receive significant support from the government through special evaluations. However, the participants in the present study were in the high-risk group and did not receive this support. Therefore, the cost burden rate is high. Hence, there is a need to increase the resilience of these individuals through economic support. Recently, the expansion of recipients of the next-highest level of out-of-pocket burden reduction support for a group with a relatively large economic burden of medical expenses was also suggested as a support plan for the medically vulnerable group [32].

The finding that depression is an influencing factor of resilience is consistent with the results of previous studies [2,10]; lower levels of depression before surgery indicate higher patient resilience. Depression can be reduced through frequent visits by the family; increased level of family support appears to lower the degree of depression. Visits can serve as social support, helping patients reduce their degree of depression. In addition, video calls from family members and acquaintances were effective in reducing depression among older residents of nursing facilities who were not allowed visitors owing to COVID-19 [33]. Visiting is an action that goes beyond a simple meeting, as it reflects support and consideration, making it an effective way to alleviate patients' depression. However, providing preoperative education and preparation of patients and their families—who constitute the primary social network— as along with educating them on post-discharge patient management, will reduce uncertainty regarding the surgical process and alleviate depression by fostering a sense of security. Moreover, family support is crucial for obstetric patients, as those undergoing obstetric surgery may experience negative self-perceptions and fear of the procedure. Encouraging positive thinking has been shown to reduce depression [34], highlighting the need for emotional support interventions that promote positive thinking among perioperative patients.

Additionally, lower levels of surgical stress indicate a higher resilience. A review of the relationship between distress and resilience in patients with brain injury and cancer [35,36] found a negative impact; this observation was consistent with the results of this study. Resilience-promoting interventions have been reported to improve positive adaptation and quality of life in patients with cancer [37]; therefore, it may be valuable to develop and evaluate interventions for surgical patients in the future. For example, physical activity helps reduce stress, improve physical and mental health, and enhance the quality of life [38-40].

Furthermore, the relationship between self-compassion and stress varies across studies. In this study, self-compassion was found to be positively related to depression and surgical stress and negatively related to social support; however, it did not impact resilience. Self-compassion was inversely correlated with isolation and depression among college students during the COVID-19 pandemic [41]. In addition, studies on female adolescents have shown that self-compassion reduces negative emotions, such as depression and anxiety [21,42]. However, previous studies examining different subsets of self-compassion have shown individuals with high levels of self-criticism appear to focus more on their mistakes or failure-related stimuli, which increases their stress and makes them more susceptible to depression [43]. Increasing self-compassion and reducing self-criticism have been suggested as effective ways to overcome these issues. Although this study did not analyze self-criticism and self-compassion separately, patients undergoing surgery are reported to be in stressful situations revealing high levels of self-criticism [30]. Therefore, the correlation between self-compassion and negative emotions such as stress and depression in patients’ preoperative needs to be investigated by categorizing self-compassion into self-compassion and self-criticism through rigorous studies. This observation suggests that although support from family and friends is important before surgery, preventing excessive emotions, such as self-compassion and self-criticism, in patients is vital in increasing resilience.

However, several limitations of this study should be considered when interpreting the results. First, as this study only selected hospital patients who visited rural areas rather than large cities, the sample may be biased. Therefore, the results of this study cannot be generalized to surgical patients in the entire community. Second, as most of the recruited participants underwent low-risk surgeries such as appendectomy, hysterectomy, ovarian removal, tendon repair, and open and closed reduction fixation, caution should be exercised when applying the results of this study to high-risk surgeries such as cancer, neurological, and cardiac surgeries. Third, the study design was cross-sectional, indicating that the data were collected at a single point in time. This factor limits their ability to identify changes in assessments over time. Longitudinal studies are beneficial for understanding stress and resilience in patients undergoing surgery. Finally, resilience is inherently subjective, and its assessment can be influenced by personal and cultural norms. Although some instruments have been validated, individual interpretations may vary. Future research is needed to test reliability and validity in surgical patients, identification of sub-factors through factor analysis, and evaluation of socio-cultural factors from multiple perspectives.

**Conclusions**

Higher education level, economic status, lower surgical stress, depression, and gynecologic surgery were found to influence surgical patients' resilience. These findings suggest that multifaceted aspects should be considered when developing interventions to improve the resilience of patients undergoing surgery. Efficient intervention programs may be developed to reduce preoperative stress and depression, particularly in obstetric and gynecological surgery. Furthermore, economic support for surgery should be expanded for vulnerable groups.

**Acknowledgments**

The authors thank the participants for making this study possible.

**Declaration of Conflicting Interests**

The authors declare no potential conflicts of interest with regarding the research, authorship, or publication of this article.

**Author Contributions**

**Conceptualization:** Heejoung Lee and Hana Ko

**Data curation:** Heejoung Lee

**Methodology:** Heejoung Lee and Hana Ko

**Software:** Heejoung Lee

**Supervision:** Hana Ko

**Writing—original draft:** Heejoung Lee and Hana Ko

**Writing—review and editing:** Heejoung Lee and Hana Ko

**References**

1.	Statistic Korea. National Health Insurance Service 2021. Available online: https://kosis.kr/statHtml/statHtml.do?orgId=350&tblId=TX_35004_A000&conn_path=I2 Accessed on 17 July, 2023.

2.	Kwak MK, Kim YK, Hong HS. Effects of abdominal breathing on preoperative anxiety and blood pressure in upper and lower extremity surgery patients. *J Korean Biol Nurs Sci.* 2011; 13(1): 23-28.

3.	Sommer M, deRijke JM, van Kleef M, Kessels AG, Peters ML, Geurts JW et al. The prevalence of post operative pain in a sample of 1490 surgical inpatients. *Eur J Anaesthesiol.* 2008;25:267–274.

4.	Jo MJ, Hong MS. The effect of supportive care in the operating room on patients' anxiety before surgery. *Korean J Adult Nurs.* 2001;13(4):632-640.

5.	Connor KM, Davidson JR. Development of a new resilience scale: The Connor‐Davidson resilience scale (CD‐RISC). *Depress Anxiety.* 2003;18(2):76-82.

6.	Davydov DM, Stewart R, Ritchie K, Chaudieu I. Resilience and mental health. *Clin Psychol Rev.* 2010;30(5):479-495.

7.	Gijzel SMW, Whitson HE, van de Leemput IA, Scheffer M, van Asselt D, Rector JL, Olde Rikkert MGM., Melis RJF. Resilience in Clinical Care: Getting a Grip on the Recovery Potential of Older Adults. *J Am Geriatr Soc*. 2019;67:2650-2657. doi:10.1111/jgs.16149

8.	Markovitz SE, Schrooten W, Arntz A, Peters ML. Resilience as a predictor for emotional response to the diagnosis and surgery in breast cancer patients. *Psychooncology.* 2015;24(12):1639-1645. doi:10.1002/pon.3834

9.	Jin Y, Bhattarai M, Kuo WC, Bratzke LC. Relationship between resilience and self-care in people with chronic conditions: A systematic review and meta-analysis. *J Clin Nurs*, 2023;32:2041– 2055.doi:10.1111/jocn.16258

10. Jeamjitvibool T, Duangchan C, Mousa A, Mahikul W. The Association between Resilience and Psychological Distress during the COVID-19 Pandemic: A Systematic Review and Meta-Analysis. *Int J Environ Res Public Health.* 2022;19:14854. doi:10.3390/ijerph192214854

11. Kim JI, Byeon YS. Factors affecting the resilience of colorectal cancer patients. *Asian Oncol Nurs*. 2013;13(4):256-264. doi:10.5388/aon.2013.13.4.256

12. Wetsch WA, Pircher I, Lederer W, Kinzl JF, Traweger C Heinz-Erian P, Benzer A. Preoperative stress and anxiety in daycare patients and inpatients undergoing fast-track surgery. *BJA*. 2009;103:199–205. doi:10.1093/bja/aep136

13. Gathinji M, McGirt MJ, Attenello FJ, Chaichana KL, Than K, Olivi A, Weingart JD, Brem H, Quinones-Hinojosa A. Association of preoperative depression and survival after resection of malignant brain astrocytoma. *Surg Neurol*. 2009;71(3):299-303. doi:10.1016/j.surneu.2008.07.016

14. Nickinson RS, Board TN, Kay PR. Post‐operative anxiety and depression levels in orthopaedic surgery: a study of 56 patients undergoing hip or knee arthroplasty. *J Eval Clin Pract*. 2009;15(2):307-310. doi:10.1111/j.1365-2753.2008.01001.x

15. O’Toole RV, Castillo, RC, Pollak AN, MacKenzie EJ, Bosse MJ. Determinants of patient satisfaction after severe lower-extremity injuries. *J Bone Joint Surg Am.* 2008;90(6):1206–1211. doi:10.2106/JBJS.G.00492

16. Vissers MM, Bussmann JB, Verhaar JA, Busschbach JJ, Bierma-Zeinstra SM, Reijman M. Psychological factors affecting the outcome of total hip and knee arthroplasty: A systematic review. *Semin Arthritis Rheum*. 2012;41(4):576-588. doi:10.1016/j.semarthrit.2011.07.003

17. Cho HM, Yoo EK. The effects of depression and family support on resilience in gynecological cancer patients. *KSW*. 2015;10(4):183-195.

18. Kaplan BH, Cassel JC, Gore S. (1977). Social support and health. *Medical care*. 1977;15(5):47-58. doi:10.1097/00005650-197705001-00006

19. Mavros MN, Athanasiou S, Gkegkes ID, Polyzos KA, Peppas G, et al. (2011) Do Psychological Variables Affect Early Surgical Recovery? PLoS ONE 6(5): e20306. doi:10.1371/journal.pone.0020306

20. Luthar SS, Cicchetti D, Becker B. The construct of resilience: A critical evaluation and guidelines for future work. *Child Dev.* 2000;71(3):543-562. doi:10.1111/1467-8624.00164

21. MacBeth A, Gumley A. Exploring compassion: A meta-analysis of the association between self-compassion and psycho-pathology. *Clin Psychol Rev.* 2012;32(6):545-552. doi:10.1016/j.cpr.2012.06.003

22. Neff KD, Kirkpatrick KL, Rude SS. Self-compassion and adaptive psychological functioning. *J Res Pers*. 2007;41(1):139-154. doi:10.1016/j.jrp.2006.03.004

23. Trompetter HR, de Klein E, Bohlmeijer ET. Why does positive mental health buffer against psychopathology? An exploratory study on self-compassion as a resilience mechanism and adaptive emotion regulation. *Cognit Ther Res*. 2017;41(3):459-468. doi:10.1007/s10608-016-9774-0

24. Kang HJ, Kim BH. A study on stress, anxiety and depression of preoperational patients. *Korean J Adult Nurs.* 1999;11(4):809-819.

25. Jeon GG, Lee MG. Korean version of CES-D development study 1. *Kor J Clin Psychol.* 1992;11(1):65-76.

26. Jeon GS, Jang SN, Park S. Social support, social network, and frailty in Korean elderly. *J Korean Geriatr Soc.* 2012;16(2):84-94. doi:10.4235/jkgs.2012.16.2.84

27. Kim KE, Lee GD, Cho YR, Chae SH, Lee WK. A Validation Study of the Korean Version of the Self-Compassion Scale: Focusing on College Students. *Kor. J. Psychol.* 2008;13(4):1023-1044. doi:10.17315/kjhp.2008.13.4.012

28. Baek HS, Lee KW, Joo EJ, Lee MY, Choi KS. *A study on the reliability and validity of the Korean Connor-Davidson ego-resilience scale* [Master’s thesis]. South Korea: Eulji University Graduate School; 2010.

29. Cohen S, Wills TA. Stress, social support, and the buffering hypothesis. *Psychol Bull.* 1985;98:310. doi:10.1037/0033-2909.98.2.310.

30. He Y, Liu X, Lin T. Guo X, Chen J. The mediating role of perceived stress in the association between family resilience and psychological distress among gynecological cancer patients: a cross-sectional study. *BMC Psychiatry.* 2024;24:622. doi.org/10.1186/s12888-024-06060-z

31. Komatsu H, Yagasaki K, Shoda R, Chung Y, Iwata T, Sugiyama J, Fujii T. Repair of the threatened feminine identity: experience of women with cervical cancer undergoing fertility preservation surgery. *Cancer nursing*. 2014;37:75-82. doi:10.1097/NCC.0b013e3182888c13

32. Hwang DK, Son BD, Park KR, Seo EW. *Research on ways to strengthen medical support of the medical vulnerable.* Sejong: Korea Institute for Health and Social Affairs;2017.

33. Lemaire C, Humbert C, Sueur C, Racin C. Use of Digital Technologies to Maintain Older Adults’ Social Ties During Visitation Restrictions in Long-Term Care Facilities: Scoping Review. *JMIR Aging.* 2023;6:e38593. doi:10.2196/38593

34. Shokrpour N, Sheidaie S, Amirkhani M, Bazrafkan L, Modreki A. Effect of positive thinking training on stress, anxiety, de-pression, and quality of life among hemodialysis patients: A randomized controlled clinical trial. *J Educ Health Promot*. 2021;30:225. doi:10.4103/jehp.jehp_1120_20.

35. Mikolajczak M, Roy E, Luminet O, De Timary P. Resilience and hypothalamic-pituitary-adrenal axis reactivity under acute stress in young men. *Stress.* 2008;11(6):477-482. doi:10.1080/10253890701850262

36. Mungase M, Chaudhury S, Patil AA, Jagtap B, Jain V. Stress, anxiety, depression, and resilience in cancer patients on radio-therapy. *Ind Psychiatry J.* 2021;30:346-352. doi: 10.4103/ipj.ipj_78_20

37. Ang WR, Ang WHD, Cham SQG, de Mel S, Chew HSJ, Devi MK. Effectiveness of resilience interventions among cancer patients–A systematic review, meta-analyses, and meta-regression of randomized controlled trials. *Eur J Oncol Nurs.* 2023;102446. doi:10.1016/j.ejon.2023.102446

38. Alvord MK, Rich BA, Berghorst LH. Resilience interventions. In: Norcross JC, VandenBos GR, Freedheim DK, Pole N, editors. APA hand book of clinical psychology: psychopathology and health. Washington, DC: American Psychological Association; 2016:505–19.

39. Ogunleye AA, Holmes MD. Physical activity and breast cancer survival. *Breast Cancer Res*. 2009;11:1-3. doi:10.1186/bcr2351

40. Mahindru A, Patil P, Agrawal V. Role of physical activity on mental health and well-being: a review. *Cureus*. 2023;15. [doi:10.7759/cureus.33475](https://doi.org/10.7759/cureus.33475)

41. Heo BY, Kim YS, Shin HJ. The relationship between college students' sense of isolation and depression in the context of COVID-19: The moderating effect of self-pity and family health. *Human Understanding*. 2022;43:129-151. doi.org/10.30593/JHUC.43.1.7

42. Barnard LK, Curry JF. Self-compassion: Conceptualizations, correlates, & interventions. Review of general psychology. 2011;15: 289-303. doi.org/10.1037/a002575

43. Cho HJ, Hyun MH. Perceived stress and self-compassion effects in self-criticism and depression. *Korean Journal of Psychology: Health*. 2011; 16: 49-62. doi.org/10.17315/kjhp.2011.16.1.004
