# Supplementary material for: Effects of preoperative stress, depression, social support, and self-compassion on resilience in surgical patients
Source: PLoS One. 2025 Apr 30;20(4):e0322609. doi: 10.1371/journal.pone.0322609 (PMC12043143; doi:10.1371/journal.pone.0322609)
Supplement: Attachment — Submitted filename: R2_Response to Reviewers.docx [file pone.0322609.s004.docx]

February 24, 2025

Kamalakar Surineni, MD, MPH

Guest Editor

PLOS ONE

Dear Editor and Reviewers:

We would like to thank you for your thoughtful comments and recommendations. We have made the following changes to the manuscript to address your concerns and revised the paper, paying particular attention to language. The revised sections of the manuscript are indicated in RED in the text.

**Reviewer 2**

| **Comments** | **Response** |
| --- | --- |
| Abstract: Authors have done a good job with dissecting the abstract into 4 separate sections. In Methods, it should say “Data was collected…” and “data was analyzed…” rather than “were”. In Results part of the abstract, it should also be clarified if the data on higher education, economic status, and gynecological surgery suggested a positive or negative correlation to resilience and if it was statistically significant or not. | As you suggested, we have revised the abstract for clarity.  Abstract  Methods  Using convenience sampling, 150 patients admitted for surgery at Nasaret International and Seoul Barun Chuckdo Hospitals in I City, South Korea were included in the study. Data was collected from July 1 to 31, 2022. Using SPSS WIN 26.0, the data was analyzed using descriptive statistics, a t-test, ANOVA, Scheffé’s test, Pearson’s correlation coefficient, and multiple regression.  Results  Surgical stress, depression, and obstetrics and gynecological surgery had statistically significant negative effects on resilience, while education level and economic status had positive effects. |
| Introduction: There are multiple grammatical errors in this section. For example, in the first para, it states that in 2021, number of patients undergoing surgical treatment will increase in 2021. However, we are already in 2025 so this statement is not accurately worded. In the second para, it states “…is perceived as major events…” but it should be “…is perceived as a major event…”. | The introduction section has once again been carefully revised, including English sentence structure and grammar.  In 2021, the number of adult surgical patients in South Korea was 1,493,000, accounting for 24% of adult inpatients (6,176,000) and 97% of all surgical patients (1,534,000), and it has continued to increase since then [1].  Undergoing a surgical procedure, even a minor one, is perceived as a major event that cause discomfort and anxiety [3,4]. |
| In the third paragraph, it states: “Resilience can be an important factor in overcoming stressful events of surgery as it affects the physical and contributes to maintaining better health. Therefore, in surgical nursing, it is important to identify and enhance the factors that influence resilience.” This section can be better worded as it appears very generic rather than worded for a study. | Resilience can be an important factor in overcoming stressful events of surgery as it affects the physical health and contributes to maintaining better health. |
| On page 4, it states “patients undergoing surgery often experience intense pressure and depression…”. Please clarify what do we mean by “intense pressure” which sounds quite ambiguous? Or do we mean stress? Also, later in that paragraph, the word “nevertheless” appears out of place.  In another part, the sentence should be modified to: “Moreover, in patients undergoing orthopedic surgery…”. | Thank you for your thoughtful review. Based on your recommendations, we have revised the sentence.  We have replaced ‘intense pressure’ with ‘anxiety’ and 'nevertheless' with 'however' to clarify the meaning.  Moreover, in patients undergoing orthopedic surgery, who require rehabilitation after surgery, the occurrence of depressive symptoms negatively affects postoperative recovery [14-16].  Nursing interventions that promote resilience by continuously assessing and managing depression should be implemented to improve the prognosis of surgical patients [17]. |
| The last paragraph of the introduction appears very convoluted and hard to read. It could be better worded for the reader. | Consequently, to better understand preoperative stress, depression, social support, and self-compassion on resilience in surgical patients, we aim to:1) determine the degree and differences in general characteristics, preoperative stress, depression, social support, self-compassion, and resilience among surgical patients; 2) examine the correlations between preoperative stress, depression, social support, self-compassion, and resilience in surgical patients; and 3) determine the impact of preoperative stress, depression, social support, and self-compassion on the resilience in surgical patients. |
| Methods: In data collection, it should read: “Data was collected after the participants…”. In Data analysis, it should read: “The data collected in this study was analyzed using IBM…” and there are other portions of the paragraph that requires the same correction in tense. | Thank you for your thoughtful review. Based on your recommendations, we have revised the Methods.  Data was collected after the participants were informed of the purpose of the study and provided written informed consent. |
| Results: The first line should read: “The patients’ sociodemographic data is presented…” Later, on page 11, it states “the total score for depression was 33.97 80” which looks like an error. On page 13, it states “However, based on previous studies, it was included and its effect on resilience was examined”. This sentence seems out of place for Results section, as it should only discuss results of the current study. Discussion about other studies can be provided in the discussion section instead. | Thank you for your thoughtful review. Based on your recommendations, we have revised the results.  We have removed the statements “However, based on previous studies, it was included and its effect on resilience was examined” in the Results section. |
| Discussion: I would recommend authors to avoid words such as “basic data” as it seems out of place in a study that is analyzing various parameters. On page 16, it states “visiting is an acting that goes beyond a simple meeting…” which can be omitted or better worded. Overall, this section provides a good discussion, including one about limitations of the study. Authors have overall done a good job in this section. However, the language / wording could be streamlined at places. | We have revised the statements, corrected the language and grammar.  This descriptive study analyzed the effects of preoperative stress, depression, social support, and self-compassion on resilience in surgical patients to provide evidence for comprehensive and personalized nursing support to aid rapid recovery after surgery. |
